# BJPsych Bulletin In Memoriam List 2024

**DOI:** 10.1192/bjb.2023.99

**Published:** 2024-02

Ademola, Adedolapo Adeniyi, Member

Anderson, Robert Moir Lechmere, Fellow ~ Over 40 Years member

Andrews, Timothy Martin, Fellow

Aryiku, Charles Amartey, Member ~ Over 40 Years member

Aslam, Masood, Member

Behfar-Rad, Mahmood, Member ~ Over 40 Years member

Belas, Reginald John, Fellow ~ Over 40 Years member

Bhattacharya, Satinath, Fellow ~ Over 40 Years member

Bradley, John Jennery, Fellow

Brown, Frank Robert Bell, Member ~ Over 40 Years member

Browne, Bernard Dominic Patrick, Fellow

Campbell, Clara, Member ~ Over 40 Years member

Campbell, Patrick Brendan, Member ~ Over 40 Years member

Clark, Iain Macdonald, Member ~ Over 40 Years member

Christie, Alexander Barlas, Fellow ~ Over 40 Years member

Cochrane, Annette, Member

Cohen, Vivienne, Fellow ~ Over 40 Years member

Cooper, Peter John, Member

Corbett, John Anthony, Fellow ~ Over 40 Years member

Daneshmand, Loghman, Member

Doig-Inglis, Lynne Louise, Affiliate

Eastwood, Jenny, Fellow ~ Over 40 Years member

Earp, James Daniel, Member

Eccleston, Donald, Fellow ~ Over 40 Years member

Ekdawi, Mounir Youssef, Fellow ~ Over 40 Years member

El Masry, Anan Abd El Aziz El Sayed, Member

Elliott, John Thomas, Fellow ~ Over 40 Years member

Evans, John, Fellow

Ghosh, Samir Kumar, Affiliate

Handa, Pratibha, Member ~ Over 40 Years member

Hanna, Gabra Elea Gabra, Member

Harbott, Anthony James, Fellow ~ Over 40 Years member

Herriott, Thompson Parker, Member ~ Over 40 Years member

Homewood, Lorna Inga Mackenzie, Fellow ~ Over 40 Years member

Johnson, Donald Arthur Wheatley, Fellow ~ Over 40 Years member

Kennedy, Robert Ian, Fellow ~ Over 40 Years member

Khan, Shaukat Ali, Member ~ Over 40 Years member

Layton, Geoffrey Peter Yonge, Member

Leyshon, Marian Wynne, Fellow ~ Over 40 Years member

Lintner, Brenda, Fellow

Marx, Clare, Honorary Fellow

Maxwell, Harold, Fellow ~ Over 40 Years member

McNamara, David Thomas, Fellow

McVitie, David Robison Craig, Fellow ~ Over 40 Years member

Meldrum, Brian Stuart, Affiliate ~ Over 40 Years member

Mir-Sepassi, Gholam Reza, Fellow ~ Over 40 Years member

Mohamed, Mohamed Bahgaat, Member

Mullan, Nuala Martina, Member

Nandi, Runa, Affiliate

Perez Gil, Antonio Juan Jose, Member ~ Over 40 Years member

Powell, Guy Alan, Member

Samuel, Michel Sadek, Fellow ~ Over 40 Years member

Sargeant, Matthew Paul, Fellow

Shafiq, Muhammad Mudassar, PMPT

Sims, Andrew Charles Petter, Honorary Fellow

Sorby, Nicholas Geoffrey Dare, Fellow

Stephens, Douglas Alan, Fellow ~ Over 40 Years member

Surendran, Gopala Pillai, Affiliate ~ Over 40 Years member

Titievskii, Alexei Vladimirovich, Specialist Associate

Tompkins, Errol Bernard, Member

Vijayasenan, Mithra Ebenezer, Member ~ Over 40 Years member

Williams, Hugh Dominic, Member

Zapata-Bravo, Enrique, Member

Zinna, Rosario-Federico, Member ~ Over 40 Years member

